# Efficacy of Biophysical Energies on Healing of Diabetic Skin Wounds in Cell Studies and Animal Experimental Models: A Systematic Review

**DOI:** 10.3390/ijms20020368

**Published:** 2019-01-16

**Authors:** Rachel Lai-Chu Kwan, Song Lu, Harry Ming-Chun Choi, Luther C. Kloth, Gladys Lai-Ying Cheing

**Affiliations:** 1 Department of Rehabilitation Sciences, The Hong Kong Polytechnic University, Hong Kong, China; rachel.lc.kwan@connect.polyu.hk (R.L.-C.K.); songlu.u.washington@gmail.com (S.L.); harry.choi@connect.polyu.hk (H.M.-C.C.); 2 Department of Physical Therapy, College of Health Sciences, Marquette University, Milwaukee, WI 53201-1881, USA; luther.kloth@marquette.edu

**Keywords:** biophysical energies, skin wounds, diabetes mellitus, cell, experimental models, systematic review

## Abstract

We have systematically assessed published cell studies and animal experimental reports on the efficacy of selected biophysical energies (BPEs) in the treatment of diabetic foot ulcers. These BPEs include electrical stimulation (ES), pulsed electromagnetic field (PEMF), extracorporeal shockwave (ECSW), photo energies and ultrasound (US). Databases searched included CINAHL, MEDLINE and PubMed from 1966 to 2018. Studies reviewed include animal and cell studies on treatment with BPEs compared with sham, control or other BPEs. Information regarding the objective measures of tissue healing and data was extracted. Eighty-two studies were eventually selected for the critical appraisal: five on PEMF, four each on ES and ECSW, sixty-six for photo energies, and three about US. Based on the percentage of original wound size affected by the BPEs, both PEMF and low-level laser therapy (LLL) demonstrated a significant clinical benefit compared to the control or sham treatment, whereas the effect of US did not reveal a significance. Our results indicate potential benefits of selected BPEs in diabetic wound management. However, due to the heterogeneity of the current clinical trials, comprehensive studies using well-designed trials are warranted to confirm the results.

## 1. Introduction

Thirty million children and adults in the United States have diabetes [1]. The incidence rate of diabetic foot ulcer is 6% [2], and 45% of diabetic patients die during the first year after the initial amputation [3]. Neuropathy, peripheral vascular disease and infection are the major risk factors for non-healing foot ulceration in patients with diabetes [4]. Increased inflammation and expression of matrix metalloprotiase-9, protein tyrosine phosphatase-1B in wound tissue and elevated level of serum growth factors were also found as the main factors associated with failure to heal diabetic foot ulcers [5]. Thus, treatments that manage neuropathy, ameliorate microcirculation and promote growth factor release may be helpful in treating chronic wounds or reducing their recurrence.

Biophysical energies (BPEs) are commonly used in physiotherapy daily practice [6]. BPE options for treating diabetic foot ulcers have included electrical stimulation (ES), MHz or kHz ultrasound (US), extracorporeal shockwave (ECSW), photo energies and pulsed electromagnetic field (PEMF). A systematic review reports positive findings on the use of the BPEs (ES, photo energies, and US) in managing foot ulcers [7] and peripheral neuropathy [8] in patients with diabetes. BPEs have been used to accelerate healing of chronic diabetic foot ulcers [9] and venous ulcers [10]. Moreover, BPEs may restore diabetes-associated microvascular [9] and neurological changes [11] that are important risk factors for delayed wound healing in patients with diabetes.

Despite the positive findings reported in some clinical studies, it is almost impossible to recruit homogeneous groups of patients in practice. Patients may respond differently to the same intervention due to variations in the severity of wound, location or chronicity. In contrast, the homogeneity in both experimental and control groups can be achieved in studies utilizing cell or animal models, and they also provide more insights into the mechanisms by which BPEs promote wound healing. Previous animal studies have shown that BPEs enhance macrophage migration [12] and antibacterial effects on ulcers [13]. In addition, BPEs have been shown to accelerate collagen deposition and enhance wound contraction in healthy Sprague-Dawley rats [14]. These animal model-based pre-clinical studies have brought some insights into the mechanisms of BPEs. However, it is important to note that rodent models cannot fully recapitulate human responses to BPEs due to mechanistic differences in wound healing, so findings from such studies may not be directly translated into clinical practice.

Thus far, there is a lack of updated review in the literature that evaluates the efficacy of BPEs for wound healing in cellular or animal models. The purpose of this review is to survey the current literature for studies that use cell culture and animal models to evaluate the efficacy of BPEs on diabetic wound healing, and to infer the underlying mechanisms of how BPEs promote wound healing.

## 2. Methods

This study followed the guidelines suggested by de Vries and co-worker [15] for reporting systematic reviews of animal studies.

### 2.1. Data Sources and Searches

The literature search for this review was restricted to published results of cellular studies and animal experiments. Databases including MEDLINE, CINAHL and PubMed were searched, covering the period from their inception to December 2018. This review was also restricted to articles published in English. Published review articles were also excluded. Keywords and Medical Subject Headings (MeSH) including PEMF, US, ECSW, ES, and LLL were combined with wound healing (limited to “cell” and “animal”) (Appendix A). A manual search of bibliographic references of relevant articles and existing reviews was also conducted to identify studies not captured by the electronic database search.

### 2.2. Study Selection

Published studies that reported the efficacy of BPEs in treating diabetic wounds were eligible for inclusion. The inclusion criteria were as follows:

- Biophysical energies
- Diabetic wound
- Cell or animal experiments

The exclusion criteria were as follows:

- Co-interventions (e.g., co-medication)
- No diabetic wounds
- Human studies
- Systematic review or meta-analysis

### 2.3. Data Extraction and Quality Assessment

Literature search was conducted independently by two reviewers (RK and MC). Articles were screened according to the title, the abstract, followed by the full paper if necessary. Duplicates were checked and removed after excluding the publications that were clearly unrelated to the purpose of this study. The full text of publications satisfying the inclusion criteria was obtained for review. At all stages, whenever there were disagreements between the two reviewers, they were resolved by discussing between themselves, sometimes with a senior and experienced reviewer (GC) or the corresponding author when necessary.

Each included experimental animal study was assessed for methodological quality by the same two reviewers independently, using SYRCLE’s risk of bias tool [16]. The checklist consists of: (1) sequence generation; (2) baseline characteristics; (3) allocation concealment; (4) random housing; (5) investigator blinding; (6) random outcome assessment; (7) assessor blinding; (8) incomplete outcome data addressed; (9) selective outcome reporting; and (10) other source of bias.

Details of the studies were extracted and summarized using a data extraction sheet. Attempts were made to obtain any missing data by contacting the authors of the studies. Data from studies published in duplicate were included only once. The data collection form consisted of demographic data (author and year published), study design characteristics (experimental groups and number of animals), animal model characteristics (species, gender, and disease etiology), intervention characteristics (dosage, timing, and duration), outcomes measures and other (dropouts).

### 2.4. Primary Outcomes

Objective measures of healing were investigated, including the healing rate of diabetic wounds, the time for complete closure, and the proportion of subjects with wound closure within the trial period.

## 3. Results

### 3.1. Search Results

**Figure 1:**
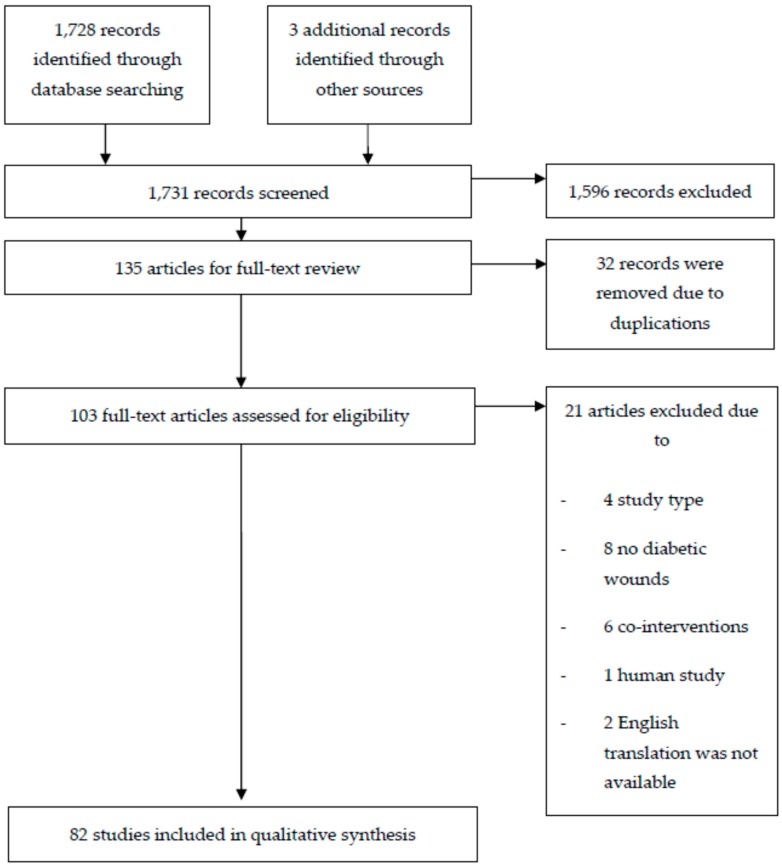
Systematic reviews flow diagram of the selected BPEs literature search.

Using the pre-defined keywords and MeSH, we identified 1731 publications pertaining to the use of BPEs for diabetic wound treatment in animal and cellular models. By screening the title and abstract, we obtained 135 relevant articles and retrieved the full text for 103 publications after removing 32 duplicated articles. Of the 103 articles, 21 were excluded for reasons related to the study design (*n* = 4), not diabetic wounds (*n* = 8), with co-interventions (*n* = 6) or human study (*n* = 1). Two articles were also not included due to the lack of English version [17,18]. Finally, 82 studies that specifically examined the effects of BPEs on diabetic wound healing were critically appraised. Figure 1 illustrates the trial selection process.

### 3.2. Characteristics of Studies

Table 1, Table 2, Table 3, Table 4, Table 5 and Table 6 present the descriptive information on each of the studies reviewed. The trials were conducted between 1984 and 2018. Overall, there were five trials on PEMF [19,20,21,22,23], three trials on US [24,25,26], four trials on ECSW [27,28,29,30], four trials on ES [31,32,33,34] and sixty-six trials on LLL [35,36,37,38,39,40,41,42,43,44,45,46,47,48,49,50,51,52,53,54,55,56,57,58,59,60,61,62,63,64,65,66,67,68,69,70,71,72,73,74,75,76,77,78,79,80,81,82,83,84,85,86,87,88,89,90,91,92,93,94,95,96,97,98,99,100]. The majority of them (60/82; 73%) were published after 2008.

### 3.3. Methodological Characteristics

The summary of methodological quality in animal studies is presented in Table 7. Only two trials have a detailed explanation of how randomization was carried out and provide an adequate report on the assignment of samples [39,46]. All trials provide baseline clinical characteristics including gender, age or weight of the subjects. In addition, all expected outcomes are reported [19,20,21,22,23,24,25,26,27,28,29,30,31,32,33,34,35,36,37,38,39,40,41,42,43,44,46,47,48,49,55,56,57,58,59,60,61,62,63,66,67,68,69,70,71,74,76,77,78,79,80,82,83,84,85,90,91,92,95,96,97,99,100]. Only one trial provides an adequate report on allocation concealment [24]. Five trials report the non-random approach when placing the animals within the facility [62,63,66,71,92]. None of the trials provide information about investigator blinding, but twenty trials report outcome assessor blinding [21,24,25,42,43,44,60,62,67,68,70,78,79,84,86,87,92,95,99,100]. Three trials report random outcome assessment, although no detailed method of randomization is provided [23,86,87]. Three trials did not include all subjects in the analysis [29,31,37]. Ten studies applied interventions to parts of the body in a single animal, accounting for the analysis bias [37,40,42,59,60,74,78,82,83,89,91].

### 3.4. Efficacy of Biophysical Energy (BPEs) Stimulation

#### 3.4.1. Pulsed Electromagnetic Field (PEMF)

The five PEMF trials compared pulsed electromagnetic fields with the sham treatment [19,20,21,22,23] (Table 1). Three trials conducted by the same researchers compared 2 mT, 5 mT and 10 mT of 25 Hz sinusoidal PEMF in male SD rats with the sham treatment [21,22,23]. One trial compared 8 mT, 20 Hz PEMF in male Wistar rats with the sham treatment [20]. Another trial involved both in vitro and in vivo studies using human umbilical vein endothelial cells, db/db mice, C57BL6 mice and FGF-2 knockout mice [19].

Wound closure percentage was the main outcome measure for all five trials. Other measures included overall wound closure time, cell proliferation, vascularity, murine endothelial cell culture, FGF-2 secretion, wound tensile strength, myofibroblast production, type 1 collagen fiber deposition, collagen fibril alignment, collagen fiber anisotropy and orientation, energy absorption capacity, Young’s modulus, wound thickness, and maximum stress of wound tissue. Four trials report significant between-group difference in the percentage of original wound size, and the experimental groups in all these studies demonstrated improved wound healing compared to the control groups [19,20,21,23].

#### 3.4.2. Ultrasound (US)

Two trials compared ultrasound with sham treatment [24,26], whereas one trial compared ultrasound with dressing changing [25]. The wound size was the main outcome measure for all three ultrasound trials. Other measures included wound closure duration, granulation tissue, collagen deposition, angiogenesis, VEGF expression, SDF-1 expression, fibroblast proliferation, speed and persistency of fibroblast migration (Table 2).

Male CD-1 mice, BKS.Cg-Dock7m+/+Leprdb/J mice, Syndecan-4 wild-type and knockout C57BL/6J mice were used in the animal models. Fibroblasts from wound tissues and db/db mouse skins were used as the cellular model. Thawer et al. and Mann et al. delivered ultrasound with saline vapor at 45 kHz and 40 kHz, respectively, while Roper et al. delivered 1 kHz ultrasound through water-based gel. Two out of three trials revealed significant between-group differences in wound size in favor of the experimental groups over the control groups in these studies [25,26]. The exception was the trial reported by Thawer and collaborators, which showed no significant between-group differences in wound size after ultrasound treatment.

#### 3.4.3. Extracorporeal Shockwave (ECSW)

Four trials on the efficacy of shock wave used male Wistar rats, SD rats, endothelial nitric oxide synthase-knockout mice, C5781/6 mice BALB/c and Bk.Cg-m Lepr (db+/db+) mice. Outcome measures included wound healing area, topical blood perfusion, leukocyte infiltration, cell proliferation, angiogenesis, wound breaking strength, collagen content, fibroblast proliferation, TGF-β1 expression in fibroblasts, myofibroblast accumulation, eNOS expression and angiogenic gene expression (Table 3).

Kuo and colleagues compared three different protocols of shockwave with the control group receiving no shockwave energy and reported a significant acceleration in wound healing (*p* < 0.05). The perfusion in wound area was significantly higher in the experimental group treated with two sessions of defocused shockwave (on postoperative Days 3 and 7) than the diabetic control group (*p* = 0.023). In addition, fibroblast count and VEGF level were upregulated in experimental groups compared to control groups. The authors concluded that treatment with an optimal session of ECSW significantly enhanced diabetic wound healing associated with increased neo-angiogenesis, tissue regeneration and topical anti-inflammatory response. However, they did not provide details on the randomization method, allocation concealment, random housing, outcome assessment, and investigator and assessor blinding [27].

Yang and colleagues compared two different protocols of shockwave with the control groups, and they reported a significant improvement evident by increased wound breaking strength, number of fibroblasts and collagen fibers. The authors concluded that low energy ECSW can improve the healing of incisional wound in diabetic rats [28]. Zins et al. investigated the angiogenic gene expressions and wound closure kinetics during diabetic wound healing with or without ECSW therapy. The expression of certain genes in the diabetic wound was augmented by shockwave, especially PECAM-1; however, they found that shockwave had no effect on wound closure in both normal and diabetic models [30].

Hayashi et al. investigated the role of endothelial nitric oxide synthase with shockwave energy for diabetic wounds. A single session of ECSW accelerated wound healing in a streptozotocin-induced diabetic mouse model, accompanied by an increased expression of eNOS and vascular endothelial growth factor (VEGF). However, the efficacy of ECSW was attenuated in eNOS-KO mice. The authors concluded that eNOS played a critical role in the therapeutic effects of shockwave by accelerating the wound healing through VEGF upregulation and neovascularization [29].

#### 3.4.4. Electrical Stimulation (ES)

The four ES trials used different types of protocols. Two trials compared ES with sham treatment [32,33]. One trial compared two different ES protocols with control receiving no ES [31]. Another trial compared ES with the control group receiving no ES or with transdermal iontophoresis by zinc sulfate [34]. None of these studies provided information about randomization, allocation concealment, investigator and assessor blinding, random housing and outcome assessment (Table 4).

Monophasic pulse wave is reported in two trials [32,33]. The outcome measures included wound healing rate, wound contraction, tensile strength, histology, collagen deposition, fibroblast proliferation and morphological analysis. Smith et al. classified the tensile strength into “poor”, “moderate” and ”good” after 10 days of stimulation, and they showed that ES enhanced diabetic wound healing. However, no statistical analysis is provided in their study [31].

Thawer et al. compared wound healing in diabetic mice with ES at 12.5 V and sham treatment (0 V). No statistical difference was found in epidermis thickness between groups. The authors suggested that ES at a high dose can alter collagen deposition in excisional wounds of diabetic mice; however, they found the effect of ES on wound healing to be disease-specific [32]. Kim and colleagues compared experimental groups receiving ES at 35–50 V with a control group receiving sham ES. Significant difference was found in wound healing rate between groups. In addition, elevated levels of collagen-I, α-SMA and TGF-β1 were found in experimental groups (all *p* < 0.05) [33].

Langoni Cassettari and collaborators divided the normal and diabetic Wistar rats into six experimental groups to study the effect of ES with direct current (DC) and zinc sulfate treatment by transdermal iontophoresis. The authors concluded that DC alone or used in association with zinc by transdermal iontophoresis was able to induce the morphological and ultrastructural changes observed during surgical wound healing in diabetic animals [34].

#### 3.4.5. Photo Energies (PE)

The photo energies reported for treating diabetic wounds encompass low-level laser energy [35,37,38,40,42,43,44,47,48,49,50,51,52,53,54,55,59,60,61,62,63,64,65,66,68,69,71,72,73,74,75,76,77,78,80,81,82,83,84,85,86,87,88,89,90,92,93,97,99,100], near-infrared [36], polychromatic light emitting diodes [39,41,45,67,70] and monochromatic infrared energy [79]. Some studies also compared different types of photo energy [46,56,57,58] (Table 5 and Table 6).

##### Low Level Laser Therapy (LLL)

A broad spectrum of laser wavelengths has been reported by different studies, whereas wavelengths in the visible red range (630–685 nm) were most commonly investigated either in isolation or in combination with other wavelengths ranging from 425 nm to 1064 nm. Power density in mWcm^2^ was not specified in some of the reviewed studies, even though this represents an important parameter. The irradiance ranged widely from 4 to 79 mWcm^2^. Peplow et al. reported a range of irradiance instead of a specific density [71]. Similarly, a large variety of animal models have been used, including C57BL/Ksj/db/db mice, SD rats, Sand rats, Wistar rats, BKS.Cg-m+/+Leprdb/J mice, Zucker diabetic rats and Swiss albino mice. Several wound healing outcomes were measured using various techniques, most commonly wound size and histology. However, nine of our surveyed trials applied laser to parts of the body of a single animal for both experiment and control, and analysis was conducted as if every single wound were from an individual animal [37,40,42,59,60,74,78,82,83,89].

##### Polychromatic Light Emitting Diodes (LED)

In six trials that investigated effects of polychromatic light emitting diodes (LED), three trials studied burn healing in diabetic rats [39,67,70]. Al-Watban et al. compared the efficacy of LED (wavelength 510–543, 594–599, 626–639, 640–670 and 842–879 nm) on burn wound at four different doses with the sham treatment. Significant burn healing was found from 48.77% to 76.77% after LED stimulation at different doses in diabetic rats [39]. The same research group also compared the efficacy of laser of different wavelengths (532, 633, 810, 980, 10600 nm) to LED clusters (510–872 nm) with incident doses of 5, 10, 20 and 30 J/cm^2^ in SD rats (*n* = 893) [56]. Their results showed that phototherapy at 633 nm should be given three times a week at a fluence of 2.35 J/cm^2^ each time for diabetic wound treatment. Wu et al. [91] compared the 635 nm laser with organic LED and showed that the organic LED significantly increased fibroblast growth factor-2 expression and macrophage activation during the initial stages of wound healing. In addition, they also found that organic LED and laser had comparative effects on promoting diabetic wound healing in rats.

##### Infrared (IR)

Danno and colleagues conducted both in vitro and in vivo studies to compare the infrared irradiation treatment with sham irradiation control or thermal control [36]. The TGF-β1 and MMP-2 content in the medium of cultured cells was significantly elevated after irradiation. Negative results in thermal controls suggested that the action of the light was athermic in nature. In animal models, the rate of wound closure was significantly accelerated after repeated exposures. Cheing and collaborators compared the efficacy of managing acute wounds in male diabetic SD rats between groups of monochromatic infrared energy (MIRE) at 890 nm and the sham group without receiving infrared energy [79]. Both experimental and sham groups showed improvement in terms of wound closure percentage; however, no statistical difference was found between groups.

## 4. Discussion

Preclinical research is important for expanding knowledge and provides insights into the cellular and physiological mechanisms on how BPEs enhance diabetic wound healing. Two trials have investigated how cells respond when exposed to electrical currents [101,102]; however, research evidence showing its effects on diabetic wound healing is limited. Four in vivo studies described here present inconsistent results regarding the value of ES in acute diabetic wound healing in animals. Thawer et al. showed no statistical difference in epidermis thickness between groups, but they did find a significant increase in collage deposition [32]. Findings reported by Kim et al. are consistent with those found by Thawer’s team, in which collagen-I expression was higher after ES. In addition, α-SMA and TGF-β1 expression were also enhanced after daily ES [33]. Langoni Cassettari et al. found accelerated wound contraction, but the morphology of inflammation was not altered after ES [34]. Statistical analysis was not available in one of the studies examined [31], making it difficult to draw conclusions on the ES’ benefits in diabetic wound healing from this animal study.

Extracorporeal shockwave (ECSW) has been used clinically for treating musculoskeletal disorders and diabetic ulcers for some years [103]. However, preclinical studies examined in this review reported contradictory findings in supporting the use of ECSW on diabetic wound healing. Two studies showed that ECSW significantly reduced wound size compared to sham treatment groups in diabetic rats [27,29]. On the contrary, a recent study by Zins et al. found that ECSW did not accelerate wound closure in wildtype (nob-diabetic) mice or db/db diabetic mice [30]. Another study found that diabetic mice treated with ECSW significantly increased the wound breaking strength and the collagen fiber content [28]. However, this effect was attenuated in endothelial nitric oxide synthase-knockout mice, suggesting that nitric oxide synthesis plays a critical role in the therapeutic effects of ECSW in diabetic wound healing [29].

Pulsed electromagnetic field (PEMF) energy has been used to treat diabetic stump wounds [104] and chronic diabetic ulcers [9]. All five studies included in our review showed positive findings that supported the use of PEMF in promotion of diabetic wound healing in animal models [19,20,21,22,23]. However, when Callaghan et al. repeated the same protocol on FGF-2 knockout mice, there was no significant improvement found in wound closure rate, suggesting FGF-2 might be a crucial factor in PEMF stimulated diabetic wound healing [19].

Sixty-six studies concerning photo energies are included in the present review. Different types of photo energies with different frequencies have been used in various studies. The wavelengths used range from visible red to infrared, power values from milliwatt to watt, and irradiation from seconds to hours. The wide range of irradiation parameters from the current review suggests the bio-modulatory potential of laser therapy [105]. In addition, these studies were conducted using various diabetic wound models, and different outcome measures were used. The findings show that irradiation by laser accelerated wound closure and collagen production, and there were increases in cellular migration, tissue viability, growth factors and gene expression. Histopathological analysis also showed a decrease in inflammatory cells and an increase in vascularization after irradiation compared to the sham control. Most trials report positive results, except Jahangiri Noudeh et al. who found no statistical significance by repeated measurements throughout the entire study period when a combined 670 nm and 810 nm laser was applied to wound areas [66]. Histological analysis revealed that there was an increase in macrophages [61,95,99], fibroblasts [47,53,63,67,68,81,84,99,100], neutrophils [95], T lymphocyte [95], collagen deposition [37,40,70,77,82,85,99,100], nitrite [100] and nitric oxide level [65], catalase activity [100], thiobarbituric acid reactive substances [100] and vascularization [44,68,70,99] after irradiation. Chung et al. adopted a splinted diabetic wound model to minimize mouse skin contraction during wound healing [62]. Seven-day treatment of 3.7–5.0 J/cm^2^ caused maximum stimulation of wound healing in diabetic mice compared to the mice receiving no irradiation. Laser irradiation of wavelength at 780 nm improved muscle repair by enhancing reorganization of myofibers and perimysium in cryoinjured diabetic rats [87]. However, not all studies demonstrated a positive result due to the specificity of absorption spectrum and laser intensity. For instance, higher frequencies might cause a negative effect on cells. Houreld and Abrahamse compared the cell morphology and expression of human IL-6 between groups receiving 5 and 16 J/cm^2^. They found that subjects treated with 16 J/cm^2^ demonstrated signs of stress without a significant increase in IL-6 expression [51]. Therefore, the optimal protocol of laser therapy for enhancing diabetic wound healing should be further investigated.

The present review does not support the use of ultrasound (US) in promoting diabetic wound healing using animal models [24,25,26]. Thawer and collaborators [24] demonstrated no significant between-group difference in wound size reduction after US, however, a significant improvement was shown by Mann et al. and Roper et al. after treatment [25,26]. Fibroblast migration and proliferation [24,25,26], as well as vascular density [24,25], were enhanced by the use of US compared to the sham groups. Interestingly, these two studies applied 40 and 45 kHz US to wounds through saline vap or or mist (as the coupling medium) for 1.5 and 3 min, respectively [24,25]. Another study utilized US at 1.5 MHz applied via traditional coupling gel for 20 min [26]. The optimal protocol for using ultrasound for enhancing diabetic wound healing should be further evaluated in future studies.

Most research on BPEs have been conducted on animal models consisting of surgically excised skin or burn wounds. However, no animal tissue model could possibly replicate the clinical situation in humans because different species may involve different healing mechanisms in skin wound, therefore, treatments with different BPEs are likely to yield different cellular responses when compared to human skin [106]. These experimental wounds excluded common problems associated with delays in healing including ischemia and infection, thus they might not present the real situation in humans [107]. In addition, Wang et al. commented that most in vitro data derived from fibroblasts of abnormal wound lesions only represent the terminal stage of the disease [107]. Therefore, these wound models may not be ideal to study the effect of BPEs on human diabetic ulcer healing. Recently, a reproducible chronic diabetic wound model that had low mortality rate was established by using Pseudomonas aeruginosa biofilm in db/db mice [108,109]. This model could be adopted in future studies to evaluate the antibiofilm effectiveness of BPEs in chronic wounds, which simulate infected diabetic ulcerations commonly seen in clinical settings. It should be noted that humane issue is always a concern of animal studies, in particular for experiments involving burn and wound. Therefore, in vitro methods might be an alternative because not only the humane concerns are circumvented but also the human cells instead of animal cells can be directly tested. Due to the shortcomings of animal studies, well-designed human studies are still the gold standard in clinical practice.

## 5. Conclusions

The present review demonstrates methodological shortcomings in animal studies that have studied the efficacy of BPEs in diabetic wound healing. One major limitation exhibited in animal experiments is that random allocation of animals to experimental and control groups and blinding is not yet a standard practice [110]. In addition, critical information for animal housing conditions and dropouts are unreported. Investigators should consider the findings of this systematic review when designing future studies and attempting to improve the internal validity of the studies by using true randomization in group allocation and outcome assessment, investigator and assessor blinding, allocation concealment, random housing, and reporting accurately on the number of animals used. In this review, the search was restricted to English publications as the translation was not available for full text review, which may have resulted in language bias. Notably, a variety of animal models were used for in vivo wound healing studies, but the physiology and healing mechanisms may not be the same across different species, and they are even more distinct compared to humans. There was considerable variation in research design, methodology, and parameters which limited comparison of research findings between studies. Therefore, findings obtained from even well-controlled animal studies may not be readily translated into clinical practice for people with diabetes management. Based on positive effects of PEMF and photo energies towards diabetic wound healing, more high-quality human clinical trials to assess the effects of those biophysical energies are warranted in the future.

## Figures and Tables

**Table 1:** Outcomes of PEMF energy for treating diabetic ulcers.

Reference	Study Type	Sample Type	Parameters	Outcome Measure	Main Results
Callaghan et al., 2008 [19]	In vivo	db/db mice (*n* = 6 in each group)	E: Asymmetric; 4.5 ms pulses; 15 Hz; magnetic flux density increased from 0 to 12 G in 200 μs and return to 0 in 24 μs; custom designed cage; 8 hrs daily C: Identical cages with inactive generators	Gross wound closure Overall wound closure time Cell proliferation Vascularity	Accelerated closure by Day 7 (E: 60 ± 5% vs C: 78 ± 6%) in db/db mice. No significant improvement in wound closure rate observed in FGF-2 knockout mice. Time to closure (E: 16 ± 4 vs C: 24 ± 5 days) in db/db mice. Higher proliferation (E: 31.5 ± 5 vs C: 7.52 ± 8 cells per high-power field) in db/db mice. Day 7 (E: 28 ± 4 vs C: 17 ± 4 cells per high power field); Day 14 (E: 32 ± 6 vs C: 21 ± 5 cells per high power field).
		C57BL6 mice (*n* = 6 in each group)			
		FGF-2 knockout mice (*n* = 6)			
	In vitro	Human umbilical vein endothelial cells	(No of plates = 6) 50 Hz inside the incubators measured less than 2 mG; harvested at each time point (0 to 12 h)	Murine endothelial cells culture FGF-2 secretion	Increased proliferation over 24 h (E: 237,876.6 ± 488 vs C: 153,386.6 ± 391 cpm). Increased after 8 h of incubation (E: 20.5 ± 6.75 vs C: 6.25 ± 0.75 cpm).
Goudarzi et al., 2010 [20]	In vivo	Male Wistar rats	E (*n* = 7): 20 Hz, 4 ms, 8 mT, 1 h/day for 10 days, with restrainer in energized coil C (*n* = 7): caged for same time without exposure to electromagnetic fields	On Days 0, 4, 8, 12, and 16 Wound healing percentage Wound healing duration Wound tensile strength	Wound healing percentage increased in treatment group more than control (*p* < 0.01). Healing time decreased in treatment group more than sham. Increased stress value in treatment group (*p* < 0.001).
Cheing et al., 2014 [21]	In vivo	Male Sprague-Dawley rats	E (*n* = 28): 5 mT, 25 Hz, 1 h daily, sinusoidal pulses, 40 ms, in plastic cylindrical container C (*n* = 28): in plastic cylindrical container without exposure to electromagnetic fields	Wound closure Myofibroblast	Increased in wound closure on Day 10 and Day 14 (E: 96.73 ± 0.4 vs C: 92.93 ± 0.57%) in treatment group (*p* < 0.05). Increased in myofibroblast on Day 7 and Day 10 in treatment group (*p* < 0.05).
Choi et al., 2016 [22]	In vivo	Male Sprague-Dawley rats	E (*n* = 20): 5 mT, 25 Hz, 1 h daily, sinusoidal pulses, 40 ms, in plastic cylindrical container C (*n* = 20): in plastic cylindrical container without exposure to electromagnetic fields	Type 1 collagen fiber deposition Collagen fibril alignment Collagen fiber anisotropy and orientation Correlation between type 1 collagen fiber abundance and myofibroblast population	Significantly greater in treatment group than control group on Day 7 (E: 0.0100 ± 0.00578 vs C: 0.00181 ± 0.000902; *p* = 0.013). No significant difference between groups No significant difference between groups Significantly more myofibroblast population on Day 7 (E: 2 ± 2 vs C: 1 ± 1; *p* = 0.042) and Day 10 (E: 4 ± 2 vs C: 2 ± 2; *p* = 0.024) in treatment group than control.
Choi et al., 2018 [23]	In vivo	Male Sprague-Dawley rats	E1: 2 mT, 25 Hz, 1 h daily E2: 10 mT, 25 Hz, 1 h daily C: in plastic restrainer bag without exposure to electromagnetic fields	Wound area Tensile biomechanical properties Wound thickness	All wounds closed by Day 14. The percent wound area of E1 was significantly smaller than C on Day 3 (*p* = 0.024). Maximum load of E2 was significantly greater than E1 (*p* = 0.012). Energy absorption capacity of E2 was significantly greater than C and E1 on Day 5 (*p* = 0.036 and 0.008 respectively). On Day 14, the Young’s modulus of E2 was significantly smaller than C (*p* = 0.023). Wound thickness of E2 was significantly greater than E1 (Day 3: *p* = 0.002) and C (Day 5: *p* = 0.014, Day 21: *p* = 0.022).

E, Experimental group; C, Control group.

**Table 2:** Outcomes of US energy for treating diabetic ulcers.

Reference	Study Type	Sample Type	Parameters	Outcome Measure	Main Results
Thawer et al., 2004 [24]	In vivo	Male CD-1 mice	E (*n* = 27): alternate days, via vapor of 15 mL prewarmed saline, perpendicular for no more than 1 cm from wound bed, 1.5 min, 5 treatments over 10 days, 45 kHz, 0.1 Watt/cm^2^ C (*n* = 23): via intravenous drip of 15 mL prewarmed saline, perpendicular for no more than 1 cm from wound bed, 1.5 min, 5 treatments over 10 days,	Wound size Granulation tissue Collagen deposition Blood vessels	Wound size in both groups decreased with no significant difference (E: 0.30 ± 0.26 vs C: 0.30 ± 0.17 cm^2^). Collagenous and vascular tissue appeared to be densely associated in the ultrasound group. Significantly greater in ultrasound group than the sham group (E: 0.92 ± 0.06 vs C: 0.82 ± 0.14). Significantly more blood vessels in the granulation tissue (E: 41.3 ± 23.0 vs C: 25.7 ± 20.3). (*p* < 0.05).
Mann et al., 2014 [25]	In vivo	Male BKS.Cg-Dock7m +/+ Leprdb /J) mice (*n* = 3 mice and *n* = 6 wound per group per time point)	E: 40 kHz with saline vapor, at distance 5 to 15 mm, 3 min, 3 times/week C: Change dressing	Wound area Wound closure duration Collagen deposition VEGF expression SDF-1 expression	On Day 9, mean wound area relative to original size decreased (E: 68 ± 3.4% vs C: 80 ± 3.2%; *p* = 0.003). Decreased wound closure duration (E: 17.3 ± 1.5 vs C: 24 ± 1.0 days; *p* < 0.05). Increased collagen deposition in ultrasound group (E: 32.8 ± 1.5 vs C: 21.0 ± 3.2; *p* < 0.05). Increased VEGF expression in ultrasound group (E: 100 ± 15.4 vs C: 41.4 ± 5.7; *p* = 0.008). Increased SDF-1 expression in ultrasound group (E: 100 ± 7.7 vs C: 53 ± 3.3; *p* = 0.003).
	In vitro	Dermal fibroblasts from db/db mice		Cell proliferation	Increased fibroblast proliferation (E: 42 ± 2 vs C: 22 ± 2; *p* < 0.001)
Roper et al., 2015 [26]	In vivo	Male Syndecan-4 wild-type; knockout C57BL/6J mice	E: 2.5 cm diameter transducer; water-based gel; 30 mWcm^−2^; 1.5 MHz; pulsed at 1 kHz, 20 min C: transducer applied but not activated	Wound size Wound closure time	Wound size significantly reduced in ultrasound group on Days 6 and 7. Wound closure time reduced 33% in ultrasound group.
	In vitro	Fibroblasts from wound tissue		Speed and persistent migration	Ultrasound switched the random migration to persistent migration in Sdc4 -/- fibroblasts.

E, Experimental group; C, Control group.

**Table 3:** Outcomes of ECSW energy for treating diabetic ulcers.

Reference	Study Type	Sample Type	Parameters	Outcome Measure	Main Results
Kuo et al., 2009 [27]	In vivo	Male Wistar Rats	E1 (*n* = 10): 1 session of defocused ESWT on postoperative Day 3 E2 (*n* = 10): 2 sessions of defocused ESWT on postoperative Day 3 and 7 E3 (*n* = 10): 3 sessions of defocused ESWT on postoperative Day 3, 7 and 10 C1 (*n* = 10): normal control without shockwave C2 (*n* = 10): diabetic control without shockwave [E1–E3: 100 impulses/area, 8 areas in all wound edges]	Wound healing time Topical blood perfusion by laser Doppler flowmetry Leukocyte infiltration by H&E staining Cell proliferation and regeneration Angiogenesis	Time course significantly reduced in E1 (8.2 ± 0.3 weeks), E2 (5.7 ± 0.6 weeks) and E3 (5.6 ± 0.4 weeks), as compared to C2 (9.8 ± 0.3 weeks) (*p* < 0.05). E2 showed significant increase in wound area perfusion compared with C2 (*p* = 0.023). Reduced in E1, E2 and E3 as compared to C1 and C2 on Day 3. Increase in fibroblasts in E1, E2 and E3 on Days 3 and 10 compared to C1 and C2. Up-regulated VEGF in E1, E2 and E3 on Day 3 and Day 10 as compared to C1 and C2.
Zins et al., 2010 [30]	In vivo	Female BALB/c, homozygous Bk.Cg-m Lepr db+/db+	E: 200 impulses, 0.1 mJ/mm^2^, 5 pulses per second, 45 s C: sham treatment	Wound closure Gene expression Angiogenesis	Shockwave does not accelerate cutaneous wound closure in wildtype normal mice or db+/db+ diabetic mice. Gene expression was augmented in both types of wound in PECAM-1 after shockwave. 44% and significant 202% increase in blood vessel density observed in shockwave-treated BALB/c and db+/db+ mice, when compared to their respective control.
Yang et al., 2011 [28]	In vivo	Male Sprague-Dawley rats	E1 (*n* = 12): 1 session of ECSW on Day 1 E2 (*n* = 12): 3 sessions of ECSW on Days 1, 3 and 5 C1 (*n* = 12): normal control without shockwave C2 (*n* = 12): diabetic control without shockwave [E1–E2: 100 impulses per cm wound length; 0.11 mJ/mm^2^; 3 Hz]	Wound breaking strength Collagen content Fibroblast proliferation TGF-β1-positive fibroblast expression	Significantly increased in E1 and E2 as compared to C2 (*p* < 0.05). Hydroxyproline content significantly increased in E1 and E2 (*p* < 0.05). Histological scores indicated ECSW-treated wounds epithelialized more rapidly and collagen fibers are more abundant at the wound site. Up-regulated significantly in E1 and E2 on Day 7 post wounding (*p* < 0.05).
Hayashi et al., 2012 [29]	In vivo	Endothelial nitric oxide synthase-knockout (eNOS-KO) mice; C578l/6 mice	E1 (*n* = 7): eNOS-KO E2 (*n* = 11): C578l/6 C1 (*n* = 6): eNOS-KO, sham C2 (*n* = 8): C578l/6, sham [E1–E2: 70.25 mJ/mm^2^; 4 Hz; 100 impulses on surface of 4 cm^2^ per side]	Wound closure Myofibroblast accumulation eNOS expression Angiogenesis	Wound closure relative to Day 0 significantly increased in E2 than C2 (88.2 ± 14.5 vs 71.1 ± 13.6%), but not in E1 and C1 (71.4 ± 12.4 vs 71.9 ± 18.6%). α-SMA-expressing myofibroblast accumulated more pronounce in E2 than C2, but did not differ between E1 and C1. eNOS increased in E2 compared to C2; CD31^+^ cells in E2 is more profound than C2 (65.9 ± 10.6 vs 50.1 ± 11.0 count/mm^2^). Vascular density significantly higher in E2 than C2 (18.9 ± 7.4 vs 10.5 ± 4.8 count/mm^2^). The difference was not seen in E1 and C1.

E, Experimental group; C, Control group; ECSW, extracorporeal shock-wave.

**Table 4:** Outcomes of ES energy for treating diabetic ulcers.

Reference	Study Type	Sample Type	Parameters	Outcome Measure	Main Results
Smith et al., 1984 [31]	In vivo	Male mice	E1 (*n* = 15): Diabetic mice, 20 volt, 20 ma E2 (*n* = 10): Diabetic mice, 1 volt, 10 ma E3 (*n* = 10): Normal mice, 20 volt, 20 ma E4 (*n* = 10): Normal mice, 1 volt, 10 ma C1 (*n* = 10): Diabetic mice, no charge C2 (*n* = 10): Normal mice without ES [E1–E3: Daily, 1 min interval, 5 days a week for 2 weeks; C1–C2: Electrode placement without charge]	Tensile strength Histology	Tensile strength in E1 and E2 is greater than C1. Longitudinal sections show restoration of hair follicles and sebaceous glands after ES than controls.
Thawer et al., 2001 [32]	In vivo	CD-1 mice (*n* = 55)	E1: Diabetic 12.5 V E2: Normal 12.5 V C1: Diabetic 0 V C2: Normal 0 V [E1–E2: restrained by flexible fiberglass narrow cone; monophasic pulsed current; pulse duration 200 ms, 200 Hz; negative electrode as treatment probe soaked in saline; 15 mins; alternate days; C1–C2: same setting except the electrode was not activated	Histology Collagen content Correlation between collagen deposition and surface area of wounds	No statistical difference found in epidermis thickness in all groups. ES energy decrease collagen amount in superficial scar in E2 as compared to C2; E1 and E2 has significantly greater collagen/non-collagenous protein ratios in deep scar than C1 and C2. Fair degrees of association between collagen deposition and surface are of wounds was found on Day 16 (*p* < 0.01).
Kim et al., 2014 [33]	In vivo	Male Sprague-Dawley rats	E (*n* = 10): diabetic rats with high voltage pulsed current stimulation daily, 100 pps, 40 min, monophasic, twin-peak pulses for 140 μs, voltage from 35 to 50 V; negative pole for first 3 days and positive for next 4 days C1 (*n* = 10): diabetic rats with sham stimulation C2 (*n* = 10): normal rats with sham stimulation	Wound healing rate Collagen-I expression α-SMA TGF-β1 mRNAs	E and C2 exhibited good wound healing as compared to C1 (*p* < 0.05). E and C2 showed significantly higher collagen-I as compared to C1 (*p* < 0.02), whereas E is highest among the groups (*p* < 0.05). E and C2 showed significantly higher α-SMA as compared to C1 (*p* = 0.04), whereas E is highest among the groups (*p* < 0.05). E and C2 showed significantly higher TGF-β1 as compared to C1 (*p* = 0.01), whereas E is highest among the groups (*p* < 0.01).
Langoni Cassettari et al., 2014 [34]	In vivo	Male Wistar rats	E1 (*n* = 20): normal with continuous ES E2 (*n* = 20): diabetic with continuous ES C1 (*n* = 20): normal without stimulation C2 (*n* = 20): diabetic without stimulation C3 (*n* = 20): normal with zinc sulfate by transdermal iontophoresis C4 (*n* = 20): diabetic with zinc sulfate by transdermal iontophoresis [E1–E2: 2 mA, 10 min; at immediate after surgical incision, Days 1, 2 and 3]	Wound contraction Fibroblasts and vascular endothelial cells proliferation Collagen fibers deposition Correlation of breaking strength and morphological findings	Wound contraction accelerated in E2 and C4 as compared to C2. Morphological inflammatory process in E2 and C4 does not differ with E1, C1, C2 and C3. Dense, progressive deposition of collagen fibers with few fenestrations on Day 4 in E2 and C4. C4 has more organizational pattern than E2. Breaking strength in C2 was significantly lower than all other groups.

E, Experimental group; C, Control group; ES, Electrical stimulation.

**Table 5:** Outcomes of in vivo studies on photo energies (PE) for treating diabetic ulcers.

Reference	Sample Type	Parameters	Outcome Measure	Main Results
**Low-level laser**				
Yu et al., 1997 [35]	C57BL/Ksj/db/db mice (*n* = 40, wound = 80)	630 nm, 20 ± 8 mW/cm^2^, 2 cm diameter, 250 s at each treatment session and received fluence of 5 J/cm^2^	Percentage of wound closure Histologic evaluation	On Day 10, significantly greater wound closure percentage in E (58.4 ± 2.6%) as compared to control (40.8 ± 3.4%). On Day 14, significantly greater wound closure percentage in E (95.7 ± 2%) as compared to C (82.3 ± 3.6%). On Day 10, significantly higher histological score in laser-treated group (6.4 ± 0.16). On Day 14, significantly higher histological score in E (12.0 ± 0.21).
Reddy et al., 2001 [37]	Male Sprague-Dawley rats	Left side wounds; 1.0 J/cm^2^ He-Ne laser at 632.8 nm; 5 days/week until wound closed	Biomechanical analysis Biochemical analyses	Maximum load and stress increased by 16%. An increase in maximum strain by 27%. No significant between-group difference found for Young’s modulus of elasticity. Energy absorption capacity increased by 47% and overall toughness increased to 84%. Total collagen for was significantly higher. There was 15% increase in neutral salt soluble collagen, 16% increase in insoluble collagen and 19% decrease in pepsin soluble collagen.
Reddy, 2003 [40]	Male Sprague-Dawley rats (*n* = 15)	Continuous infrared radiation at 904 nm produced by Ga-As laser, 7 mW, 1.0 J/cm^2^, once a day, 5 days/week until wound closed	Biomechanical analysis Biochemical analyses Hydroxyproline Collagen maturation	Significant increase in tensile strain. Marginal increases were observed in tensile strength and stress indices. Total collagen increased by 14%. Collagen deposition increased. Insoluble collagen increased by 50%.
Danno et al., 2001 [36]	Male ICR mice (*n* = 20); Female C57BL/KsJ-db/db mice (*n* = 20)	Daily, 30 min, at distance of 20 cm, 54 J/cm^2^	Wound area	The rate of wound closure significantly accelerated.
Stadler et al., 2001 [38]	C57BL/Ksj/db/db mice; Heterozygous littermates as control (*n* = 20)	Class IIIb 830 nm laser; 79 mW/cm^2^, daily, 5 J/cm^2^/wound; 5 consecutive days; 0–4 days or 3–7 days	Tensile strength	Tensile strength at 11 days was significant between diabetic laser group (2.16 ± 0.47 g/mm^2^) and sham (1.28 ± 0.32 g/mm^2^). Tensile strength at 23 days E than in C (2.72 ± 0.56 g/mm^2^ vs 1.5 ± 0.3 g/mm^2^).
Byrnes et al., 2004 [42]	Psammomys obesus (Sand rats)	Diabetic, 4 J/cm^2^, He-Ne gas laser: 632.8 nm, daily for 3 consecutive days, at left wound	Wound closure percentage Histological characteristics	Wound closure was significantly faster in E (34.3 ± 10.5, 68.4 ± 10.4%) than C (−42.0 ± 23.3, 28.5 ± 10.8%) on Days 2 and 10. Three-fold increase in bFGF in E as compared to C.
Kawalec et al., 2004 [43]	C57BLKS/J mice (*n* = 56)	E1: 5 W every 2 days, 18 J/cm^2^ E2: 5 W every 4 days, 18 J/cm^2^ E3: 10 W every 2 days, 36 J/cm^2^ E4: 10 W every 4 days, 36 J/cm^2^ GaAIAs diode laser, 980 nm, 1 s	Wound closure percentage Histological characteristics	Wound closure percentage was only significant in E2 and E3 on Day 5. Average score of 5.8 on Day 7 and 15.5 on Day 14 in E1.
Maiya et al., 2005 [44]	Male Wistar rats (*n* = 48)	632.8 nm, 4.8 J/cm^2^, He-Ne laser, 5 days per week until closed	Biochemical analysis Histopathological analysis	Total collagen for E was significantly higher. Significant increase in fibroblastic proliferation, capillary proliferation, granulation tissue formation, vascularity and epithelization on Day 4.
Carvalho et al., 2006 [47]	Male Wistarrats	632.8 nm HeNe laser, 4 J/cm^2^, 60 s/wound, continuous, 5 mW	Histology	Significant difference in collagen.
Rabelo et al., 2006 [48]	Male Wistar rats (*n* = 50)	3 times/week, continuous, 632.8 nm HeNe laser, 10 J/cm^2^, 17 s	Qualitative histopathological analysis Quantitative histological analysis	Less intense inflammatory process Significant decrease of the inflammatory cell density and significant increase in capillarity.
AI-Watban et al., 2007 [49]	Male Sprague-Dawley rats (*n* = 52)	E1: 532 nm, 5 J/cm^2^ E2: 633 nm, 5 J/cm^2^ E3: 810 nm, 5 J/cm^2^ E4: 980 nm, 5 J/cm^2^ E5: 532 nm, 10 J/cm^2^ E6: 633 nm, 10 J/cm^2^ E7: 810 nm, 10 J/cm^2^ E8: 980 nm, 10 J/cm^2^ E9: 532 nm, 20 J/cm^2^ E10: 633 nm, 20 J/cm^2^ E11: 810 nm, 20 J/cm^2^ E12: 980 nm, 20 J/cm^2^ E13: 532 nm, 30 J/cm^2^ E14: 633 nm, 30 J/cm^2^ E15: 810 nm, 30 J/cm^2^ E16: 980 nm, 30 J/cm^2^	Wound healing percentage	The percentage of wound healing acceleration is higher in all treatment groups than the control groups. The optimum wavelength and incident dose was at E6.
Meireles et al., 2008 [55]	Male Wistar rats (*n* = 55)	E1: 660 nm, 20 J/cm^2^ E2: 780 nm, 20 J/cm^2^	Histology	At Day 7, E1 as necrosis extended down to epidermis, and E2 has extending down to dermis. On Day 14, E1 and E2 showed moderate amount of neo-angiogenesis. On Day 21, E1 showed advanced re-epithelialization, but E2 showed no epithelialization.
Gungormus and Akyol, 2009 [59]	Female Wistar rats	Class IV, medical class IIB, 20 W, 50 Hz, GaA1As 808 nm, continuous, 0.1 W/cm^2^, 10 J/cm^2^, on Days 2, 4, 6, and 8	Degree of re-epithelialization and inflammation	Significant between-group difference was found in re-epithelialization and inflammation on Day 10, but not on Day 20.
Akyol and Gungӧrmuş, 2010 [60]	Wistar rats (*n* = 54)	Diode laser; 808 nm, 0.1 W/cm^2^, Day 0,2,4,6 and 8, 10 J/cm^2^, 20 s per session	Histology analysis	Significant difference found in post hoc analysis between E and C in re-epithelialization and inflammation on Day 10.
Carvalho pde et al., 2010 [61]	Male Wistar rats	InGaA1P diode laser, continuous, 100 mW, 660 nm, 10 J/cm^2^	Histology analysis Morphometric analysis	Significant difference in mean collagen between E and C (19.96 ± 1.89 vs 13.19 ± 3.70; *p* = 0.0457) on Day 3, and on Day 5 (30.95 ± 4.14 vs 16.95 ± 2.36). Significant difference was found in mean number of macrophages between E and C on Day 3, Day 5 and Day 7.
Chung et al., 2010a [63]	BKS.Cg-m+/+Leprdb/J (*n* = 47)	E1: 660 nm, 20 s, 18 mW, 7 consecutive days, 0.36 J/day E2: 660 nm, 20 s, 80 mW, 7 consecutive days, 1.6 J/day	Wound area Histological analysis	E2 increased the mean wound area on Day 4, but decreased in wound area on Day 14 as compared to E1 and C. The mean dermal gap and epithelial gap for E2 was significantly different from C but not E1.
Chung et al., 2010b [62]	BKS.Cg-m+/+Leprdb/J	E1: 660 nm, 0 s, 80 mW, 7 consecutive days, 0 J/day E2: 660 nm, 10 s, 80 mW, 7 consecutive days, 0.8 J/day E3: 660 nm, 20 s, 80 mW, 7 consecutive days, 1.6 J/day E4: 660 nm, 30 s, 80 mW, 7 consecutive days, 3.2 J/day	Histological analysis	In splinted wound, the mean dermal gap and epithelial gap for E3 was significantly different from E1, 2 and 3. All wounds in E3 completely re-epithelized, and granulation tissue with collagen fibers filled or almost filled the whole of wound bed in splinted wound.
Jahangiri Noudeh et al., 2010 [66]	Male Wistar rats (*n* = 19)	GaA1InP laser, 670 nm, 10 J/cm2; combined with 810 nm GaA1As laser, 250 mW, 12 J, 50 s, 1.33 J/cm^2^, performed every 3 days	Wound area	No statistical significance in wound area throughout repeated measurements in the study time period.
Santos et al., 2010 [68]	Male Wistar rats (*n* = 12)	E1: 680 nm, 40 J/cm^2^ per session E2: 790 nm, 40 J/cm^2^ per session	Histological analysis	Fibroblast number and angiogenesis was higher in E2. Necrosis was more evident in E1.
Hegde et al., 2011 [69]	Male Swiss albino mice	E1: 4 min, 15 s^−1^ J cm^−2^ E2: 8 min, 32 s^−2^ J cm^−2^ E3: 12 min, 46 s^−3^ J cm^−2^ E4: 17 min, 3 s^−4^ J cm^−2^ E5: 21 min, 17 s^−5^ J cm^−2^ [E1–E5: 632.8 nm HeNe laser]	Biochemical analysis	Hydroxyproline content in granulation tissue on Day 6 and Day 12 revealed a significant increase in the collagen content in all treatment groups. Rise in glucosamine levels was observed in all experimental groups on Day 6 but subsequently decreased linearly.
Peplow et al., 2011 [71]	BKS.Cg-m+/+Leprdb/J	E1: 100 mW, 233–313 mW/cm^2^ E2: 50 mW, 116–156 mW/cm^2^ E3: 25 mW, 58–78 mW/cm^2^ [E1–E3: 660 nm]	Histological analysis	All splinted wounds were completely re-epithelized, and granulation tissue with collage fibers filled or almost filled the whole wound bed.
Dadpay et al., 2012 [74]	Male Wistar rats (*n* = 18)	0.2 J/cm^2^, pulsed infrared diode laser, 1.08 W/cm^−2^, 890 nm, 80 Hz	Biomechanical examination	Significant increases in maximum load and accelerate wound healing.
Park and Kang, 2012 [89]	Male Sprague-Dawley rats (*n* = 48)	980 GaA1As diode laser, 60 s every day, 0.01 W, 13.95 J/cm^2^	Histological analyses Gene expression	Histological observations and gene expression analyses revealed a faster initial healing and more alveolar bone formation.
Peplow et al., 2012 [76]	BKS.Cg-m+/+Leprdb/J	660 nm, 100 mW, 20 s/day, 7 days	Body weight and water intake Glucose and GHbA1c levels in blood plasma	There were no significant differences in body weight and water intake over 22 days. On Day 14, the mean blood plasma glucose level was not significantly different between E and C. GhbA1c was not detected.
Aparecida Da Silva et al., 2013 [77]	Male Wistar rats (*n* = 120)	InGaA1P, 50 mW, 660 nm, 4 J/cm^2^	Histological analysis Morphometric analysis MMP-2 and MMP-9 synthesis	The density of total collagen of E was significantly higher than C. Collagen I was always greater than that observed in collagen III in all groups. Significant increase in MMP-2 and MMP-9 expression in C than E.
Fathabadie et al., 2013 [78]	Male Wistar rats (*n* = 72)	Once daily for 6 days a week, pulsed infrared laser, 75 W, 1.08 W/cm^2^, 890 nm, 80 Hz, 180 ns pulse duration, 200 s, 0.2 J/cm^2^	Morphometric examination	Significantly increased the number of mast cells on Days 4 and 15 after surgery.
Firat et al., 2013 [86]	Male Wistar rats (*n* = 42)	GaA1As laser, 940 nm, 10 J/cm^2^, 0.1 W, continuous for 9 s, first dose at 2 h after wounding, then at 2 days interval for 4 sessions	Histological analysis Biochemical analysis	Histopathological findings revealed a decrease in number of inflammatory cells, and increased mitotic activity of fibroblasts, collagen synthesis, and vascularization. The total oxidative status was significantly deceased on Day 21.
Franca et al., 2013 [87]	Male Wistar rats (*n* = 65)	780 nm, 5 J/cm^2^, 10 s/point, 0.2 J	Morphologic evaluation Collagen analysis Muscle fiber area	On Day 14, E was in the remodeling phase, C was still in the proliferative phase, with fibrosis, chronic inflammation, and granulation tissue. Under polarized light, on Day 14, E had organized collagen bundles in the perimysium. C exhibited more myonecrosis than E.
Dancáková et al., 2014 [80]	Male Sprague-Dawley rats (*n* = 21)	810 nm laser	Tensile strength Histological evaluation and morphometry	Reduced the loss of tensile strength and increased the wound stiffness significantly. Significantly more mature granulation tissue.
Kilík et al., 2014 [82]	Male Sprague-Dawley rats (*n* = 48)	GaA1As 635 nm, three times daily, 5 J/cm^2^; 1st wound: 1 mW/cm^2^; 2nd wound: 5 mW/cm^2^; 3rd wound: 15 mW/cm^2^	Histopathological evaluation	The synthesis and organization of collagen fibers were consecutively enhanced in the 15 mW/cm^2^ group. A significant difference in the number of newly formed capillaries.
Sharifian et al., 2014 [83]	Male Wistar rats (*n* = 24)	890 nm, 6 days per week, pulsed infrared laser, 80 Hz, 0.2 J/cm^2^	Histomorphometry bFGF gene expression	Laser significantly increased the numbers of macrophages, fibroblasts, and blood vessel sections. bFGF expression at 48 h revealed a significant increase in gene expression.
De Loura Santana et al., 2015 [84]	Female Wistar rats (*n* = 90)	E1: laser 1 J/cm^2^, 26 s, 4 times E2: laser 4 J/cm^2^, 26 s, 1 time Gallium-aluminum-arsenide diode laser, 660 nm	Wound closure rate Healing morphology, inflammatory infiltrate and myofibroblasts count Collagen deposition and optical retardation of collagen	Laser accelerated wound closure by 40% in first 3 days. Laser increased acute inflammatory infiltrate until Day 3. More myofibroblasts and better collagen organization.
Lau et al., 2015 [85]	Male Sprague Dawley rats (*n* = 120)	E1: 100 mW, 50 s, 0.1 W/cm^2^ E2: 200 mW, 25 s, 0.2 W/cm^2^ E3: 300 mW, 17 s, 0.3 W/cm^2^ 808 nm diode laser, continuous mode, 5 J/cm^2^, once daily	Wound contracture Histology	The wound contracture was found optimized. Laser therapy enhanced epithelialization and collagen fiber synthesis.
Lau et al., 2015 [90]	Male rats (*n* = 21)	E1: 110 mW, 30 s E2: 110 mW, 60 s E3: 110 mW, 120 s E4: 510 mW, 30 s E5: 510 mW, 60 s E6: 510 mW, 120 s 808 nm diode laser, continuous mode	Tensile strength	Tensile strength in E4–E6 enhanced as compared to control and E1–E3.
Fekrazad et al., 2015 [92]	Male Wistar rats (*n* = 40)	E1: blue (425 nm) laser, 50 mW/cm^2^, 2 J/cm^2^ E2: green (532 nm) laser, 55 mW/cm^2^, 2 J/cm^2^ E3: red (630 nm) laser, 50 mW/cm^2^, 2 J/cm^2^	Wound healing	Significant difference in the mean slope of wound healing between E and C.
de Loura Santana et al., 2016 [95]	Female Wistar rats (*n* = 90)	E1: Single dose laser, 4 J/cm^2^, 104 s, 3.12 J, Day 1 E2: Fractionated-dose laser, 1 J/cm^2^, 26 s, 0.78 J, Days 1, 3, 8 and 10 660 nm, 30 mW, 38 mW/cm^2^	Immunohistochemistry Inflammatory cell counts	Neutrophils were predominant in E1 on Day 1. E1 exhibited greater number of total macrophages on Day 3. CD206+ cell counts revealed that E1 had more M2 macrophages on Day 8, whereas E2 exhibited more M2 macrophages on Day 10.
Ranjbar et al., 2016 [99]	Male Wistar rats (*n* = 30)	685 nm InGaA1P laser, 15 mW, 3 J/cm^2^, 0.028 cm^2^	Bacterial growth Wound length Histological structures Breaking strength	Mean bacterial numbers (0.51 × 10^1^ ± 0.2 × 10^1^ CFU/mL) were significantly lower). Length of wounds in E were significantly shorter on Days 14 and 21. Significant increase in number of macrophages and new blood vessels, and also significant elevated fibroblast number and collagen deposition. E significantly increased in breaking strength.
Tatmatsu Rocha et al., 2016 [100]	Male Swiss mice (*n* = 20)	904 nm GaAs diode laser, 5 days, 40 mW, 60 s	Histopathological analysis Collagen amount Catalase activity Nitrite Thiobarbituric acid reactive substances	Moderate amount of fusiform fibroblasts, an increased density of blood vessels and intense deposition of a more organized collagen matrix was observed. Significant differences in type II fibers. Higher catalase activity. Decreased concentration of nitrite and nitrite concentration compared. Significantly lower levels of thiobarbituric acid reactive substances.
Denadai et al.,2017 [96]	Wistar rats (*n* = 36)	660 nm InGaAlP, 100 mW, 60 s, 6 J/cm^2^, 0.028 cm^2^	Malondialdehyde levels	Significant lower level of malondialdehyde.
Eissa and Salih, 2017 [97]	Wistar rats (*n* = 14; 6 males, 8 females)	632.8 nm He-Ne laser, continuous, aperture ~2.3 × 10^−6^ mm, 4 mW/cm^2^, 4 min, 6 mm away from skin, 5 days/week until wound healed	Wound diameter	E healed on average on Day 21, whereas C healed after 40 days of 60 days.
**Polychromatic light emitting diodes (LED) energy**				
AI-Watban and Andres, 2003 [39]	Sprague-Dawley rats (*n* = 30)	E1: 5 J/cm^2^ E2: 10 J/cm^2^ E3: 20 J/cm^2^ E4: 30 J/cm^2^ 25-LED array (510–543 nm; 594–599 nm; 626–639 nm; 640–670 nm; 842–872 nm); 13.6 mW/cm^2^; 3 times/week; 3 consecutive weeks	Healing rate	Healing accelerated at 5 and 10 J/cm^2^, but no significant inhibition seen at 20 and 30 J/cm^2^.
Whelan et al., 2003 [41]	BKS.Cg-m +/+Leprdb (*n* = 80)	670 nm LED with restrainer; daily for 14 days; 4 J/cm^2^; 28 mW/cm^2^ for 2 min and 24 s	Wound healing rate RNA	Wound healing rate increased. Galectin-7 is upregulated at Day 2 and continued to be elevated after 14 days of treatment. Fibroblast growth factor 7 and 12 were upregulated by 2 days. Genes of TGF-Beta 1 and thrombospondin 1 were upregulated by 14 days of treatment.
Oliveira et al., 2010 [67]	Male Wistar rats (*n* = 30)	E1: Polarized light 400–2000 nm, 20 J/cm^2^ E2: Polarized light 400–2000 nm, 40 J/cm^2^	Histological analysis	Significant difference in revascularization and re-epithelialization.
Oliveira et al., 2011 [70]	Male Wistar rats (*n* = 90)	E1: polarized light 400–2000 nm, 10.2 J/cm^2^ E2: polarized light 400–2000 nm, 20.4 J/cm^2^	Histological analysis	10.2 J/cm^2^ caused higher deposition of collagen, quicker inflammatory reaction and improved revascularization than 20.4 J/cm^2^.
**Monochromatic infrared energy (MIRE)**				
He et al., 2013 [79]	Male Sprague-Dawley rats (*n* = 30)	890 nm, intensity set at level 6, 85% of full power, 30 min, three times a week before euthanized	Wound closure percentage Histological analysis	No significant difference was found between E and C for would closure. No significant difference was found between E and C for re-epithelialization, cellular content, myofibroblast population and granulation tissue formation at each time point. Greater deposition of type I collagen was found in E as compared to C at end of Week 2.
**Comparing different photo energies**				
AI-Watban and Andres, 2006 [46]	Male Sprague-Dawley rats (*n* = 61)	E1: 5 J/cm^2^ E2: 10 J/cm^2^ E3: 20 J/cm^2^ E4: 30 J/cm^2^ 25-LED array (510–543 nm; 594–599 nm; 626–639 nm; 640–670 nm; 842–872 nm); 13.6 mW/cm^2^; 3 times/week; 3 consecutive weeks	Wound healing percentage	Wound healing percentage was significant for E1 (16 ± 3.1%, *p* = 0.01) but not significant for E2, 3 and 4 (7 ± 3.4, 3.4 ± 3.5, 0.9 ± 3.6%).
AI-Watban, 2009 [56]	Sprague-Dawley rats (*n* = 893)	E1: 5 J/cm^2^ E2: 10 J/cm^2^ E3: 20 J/cm^2^ E4: 30 J/cm^2^ [E1–E4: laser 532 nm, 143 mW, 20.4 mW/cm^2^] E5: 5 J/cm^2^ E6: 10 J/cm^2^ E7: 20 J/cm^2^ E8: 30 J/cm^2^ [E5–E8: laser 633 nm, 140 mW, 15.56 mW/cm^2^] E9: 5 J/cm^2^ E10: 10 J/cm^2^ E11: 20 J/cm^2^ E12: 30 J/cm^2^ [E9–E12: laser 810 nm, 200 mW, 22.22 mW/cm^2^] E13: 5 J/cm^2^ E14: 10 J/cm^2^ E15: 20 J/cm^2^ E16: 30 J/cm^2^ [E13–E16: laser 980 nm, 200 mW, 22.22 mW/cm^2^] E17: 5 J/cm^2^ E18: 10 J/cm^2^ E19: 20 J/cm^2^ E20: 30 J/cm^2^ [E17–E20: laser 10,600 nm, 300 mW, 66.37 mW/cm^2^] E21: 5 J/cm^2^ E22: 10 J/cm^2^ E23: 20 J/cm^2^ E24: 30 J/cm^2^ [E21–E24: Polychromatic LEDs 510–872 nm, 272 mW, 13.6 mW/cm^2^] [three times per week]	Wound area	The best effects on wound healing was shown in E5–E8, followed by E1–E4 > E13–E16 > E9–E12 > E21–E24 > E17–E20.
AI-Watban et al., 2009 [57]	Male Sprague-Dawley rats	E1: 5 J/cm^2^ E2: 10 J/cm^2^ E3: 20 J/cm^2^ E4: 30 J/cm^2^ [E1–E4: laser 532 nm, 143 mW, 20.4 mW/cm^2^] E5: 5 J/cm^2^ E6: 10 J/cm^2^ E7: 20 J/cm^2^ E8: 30 J/cm^2^ [E5–E8: laser 633 nm, 140 mW, 15.56 mW/cm^2^] E9: 5 J/cm^2^ E10: 10 J/cm^2^ E11: 20 J/cm^2^ E12: 30 J/cm^2^ [E9–E12: laser 670 nm, 120 mW, 22.86 mW/cm^2^] E13: 5 J/cm^2^ E14: 10 J/cm^2^ E15: 20 J/cm^2^ E16: 30 J/cm^2^ [E13–E16: laser 810 nm, 200 mW, 22.22 mW/cm^2^] E17: 5 J/cm^2^ E18: 10 J/cm^2^ E19: 20 J/cm^2^ E20: 30 J/cm^2^ [E17–E20: laser 980 nm, 200 mW, 22.22 mW/cm^2^] [three times per week]	Relative healing	Significant difference in the mean percentage of healing acceleration between the visible laser and invisible laser.
Dall Agnol et al., 2009 [58]	Male Wistarrats	E1: GaA1As LED, 40 nm bandwidth centered at 640 nm, 30 mW E2: indium-gallium-aluminum-phosphide (InGaA1P) laser, 660 nm, 30 mW, 6 J/cm^2^	Wound diameter Microscopic evolution Qualitative microscopic analysis	Significantly reduced in wound diameter: 45% in E1 and 44.5% in E2. The number of inflammatory cells in E1 and E2 was reduced by 23% at the shallow dermis region, and 19% in the deep dermis. Histological characteristics indicated an acceleration of the cicatrization process by the phototherapy.
Wu et al., 2015 [91]	Male Zucker Diabetic Fatty rats (*n* = 30)	E1: Organic light-emitting diode E2: 635 nm laser [10 mW/cm^2^, 5 J/cm^2^, 8 mins 20 s, Daily for 7 consecutive days]	Wound closure measurement Histological score Immunohistochemistry	Percentage wound closure significantly higher in E1 (40.94 ± 3.49%). E1 and E2 had significantly higher histological scores. Significantly higher level of FGF2 expression.

E, Experimental group; C, Control group.

**Table 6:** Outcomes of in vitro studies on photo energies for treating diabetic ulcers.

Reference	Sample Type	Parameters	Outcome Measure	Main Results
**Low-level laser**				
Houreld and Abrahamse, 2007a [50]	Human skin fibroblast cells	E1: 26 min 33 s, 5 J/cm^2^ E2: 84 min 23 s, 16 J/cm^2^ Exposed once on Days 1 and 4, HeNe laser 632.8 nm, 3 mW/cm^2^	Cell morphology Cytotoxicity Apoptosis Genetic integrity	No marked morphological changes were observed in cells following laser. Exposure of E1 did not induce additional damage to cells; Exposure to E2 significantly increased amount of cellular lysis. Apoptosis was significantly increased. Additional DNA damage was not seen in E1, but in E2.
Houreld and Abrahamse, 2007b [51]	Human skin fibroblast cells	E1: 37 min, 5 J/cm^2^ E2: 2 h, 16 J/cm^2^ HeNe laser 632.8 nm, 2.206 mW/cm^2^	Cell morphology Expression of human IL-6 Neutral red assay	No marked morphological changes were observed in E1; cells in E2 showed sign of stress with open spaces. Significant increase in human IL-6 in E1, but no significant changes in E2. Significant increase in neutral assay in, significant decrease in neutral assay was shown in E2.
Houreld and Abrahamse, 2007c [52]	Human skin fibroblast cells	E1: 27 min 46 s, 5 J/cm^2^, at 30 min and 24 h E2: 2 h, 16 J/cm^2^, at 30 min and 72 h HeNe laser 632.8 nm, 3.034 mW/cm^2^	Cell morphology Cell viability Cytotoxicity and genetic integrity	E1 and E2 showed more chemotaxis and haptotaxis at 30 min. No significant change in percentage of ATP viability in E1 and E2 after 30 min. Decrease in viability at E1 at 24 h. Significant increase in cytotoxicity and DNA damage in E1 and E2 after 30 min. Significant damage in DNA seen at 24 h and 72 h in E1 and E2.
Mirzaei et al., 2007 [53]	Cultures of fibroblast-like cells from Wistar rats	E1 (wells *n* = 10): 0.09 J/cm^2^, 30 s, 4 times/day E2 (wells *n* = 10): 0.09 J/cm^2^, 30 s, 4 times at 2 days E3 (wells *n* = 10): 1 J/cm^2^, 330 s, 4 times at 2 days E4 (wells *n* = 10): 1 J/cm^2^, 100 s, 4 times at 4 days E5 (wells *n* = 10): 4 J/cm^2^, 1320 s, 4 times at 4 days [E1–E5: HeNe laser 632.8 nm, 0.6 mW]	Viability Number of cells Transmission electron microscopy	More bipolar and spindle-shaped fibroblasts in the laser-treated cultures than in the sham-exposed. Significant increase in the number of cells in E5. Ultrastructure features of fibroblasts in the sham-exposed and laser-treated cultures were similar.
Houreld and Abrahamse, 2008 [54]	Human skin fibroblast cells	E1: HeNe 632.8 nm, 5 J/cm^2^, 23 mW, 2.206 mW/cm^2^ E2: HeNe 632.8 nm, 16 J/cm^2^, 23 mW, 2.206 mW/cm^2^ E3: diode 830 nm, 5 J/cm^2^, 55 mW, 6 mW/cm^2^ E4: diode 830 nm, 16 J/cm^2^, 55 mW, 6 mW/cm^2^ E5: Nd:YAG 1064 nm, 5 J/cm^2^, 1 W, 12.7 mW/cm^2^ E6: Nd:YAG 1064 nm, 16 J/cm^2^, 1 W, 12.7 mW/cm^2^	Morphology Cellular viability Cellular proliferation	The rate of cellular migration into the central scratch was significantly higher in E1 than C. Cells radiated at E3 showed more migration into the central scratch compared to E4. E4 and E5 did not show an increased rate of cellular migration. E1 showed a significant increase in percentage viability compared to E2. Cells radiated with E2 showed a decrease in percentage viability but was not significant. Cells radiated in E3, E4, E5 and E6 show no significant change in percentage viability. E1 and E3 showed a significant increase in bFGF expression.
Houreld and Abrahamse, 2010 [64]	Human skin fibroblast cells	E1: HeNe 632.8 nm, 5 J/cm^2^, 23 mW, 2.206 mW/cm^2^ E2: diode 830 nm, 5 J/cm^2^, 55 mW, 6 mW/cm^2^ E3: Nd:YAG 1064 nm, 5 J/cm^2^, 1 W, 12.7 mW/cm^2^	Morphology Cellular viability Cellular proliferation	E3 showed less migration into the central scratch and incomplete wound healing. E1 and E2 showed higher rate of migration and haptotaxis with complete wound closure. No significant change in ATP luminescence in E1 and E2, whereas E3 showed a significant decrease to all other groups. Significant increase in bFGF in E1 and E2.
Houreld et al., 2010 [65]	Human skin fibroblast cells (*n* = 6)	830 nm, 40 mW, 5 J/cm^2^	Cellular viability Apoptosis Cellular proliferation Cytokine expression Nitric oxide Reactive oxygen species	No significant change in viability A decrease in apoptosis 24 h post irradiation. Significant increase in proliferation at 24 and 48 h. TNF-α were significantly decreased at both 1 and 24 h. No significant change in IL-6. An increase in NO 15 min post irradiation. An increase in ROS 15 min post irradiation.
Sekhejane et al., 2011 [72]	Diabetic wounded and hypoxic human skin fibroblast cells (WS1)	636 nm, continuous, 5 J/cm^2^, 476 s and incubated for 1 or 24 h	Cellular morphology Viability Apoptosis Proliferation Cytokine expression	Regained in cellular morphology. Increase in cellular viability. Decrease in apoptosis. All cells model showed an increase in proliferation. Decrease in TNF-α and proinflammatory cytokine interleukin IL-1β. E3 showed a decrease in TNF-α.
Ayuk et al., 2012 [73]	Diabetic wounded human skin fibroblast	660 nm, continuous, 10.22 mW/cm^2^, 5 J/cm^2^, 8 min 9 s and incubated for 48 or 72 h	Cellular morphology Cellular viability Cellular proliferation Collagen-I	Significant increase in cell migration, viability, proliferation and collagen production.
Houreld et al., 2012 [75]	Human skin fibroblast	E1: 5 J/cm^2^ E2: 15 J/cm^2^ 660 nm, continuous, 11 mW/cm^2^	Enzymatic activities ATP luminescent assay Mitochondrial staining	E2 showed a significant decrease in complex III activity. ATP showed a significant increase in E2. There are higher accumulations of active mitochondria.
Esmaeelinejad et al., 2014 [81]	Human skin fibroblasts	E1: 757 s, 0.5 J/cm^2^ E2: 1512 s, 1 J/cm^2^ E3: 3024 s, 2 J/cm^2^ HeNe laser, 1.5 mW, 632.8 nm, 0.66 mW/cm^2^	Cell morphology Proliferation rate and cell viability	Biological changes in cell morphology were clearly visible in laser-treated human skin fibroblasts at energy densities of 0.5, 1 and 2 J/cm^2^. Laser delivered at densities of 0.5 and 1 J/cm^2^ had stimulatory effects on the viability and proliferation rate of human skin fibroblasts cultured in physiologic glucose concentration.
Masha et al., 2013 [88]	Human skin fibroblast cells (WS1)	660 nm, continuous, 100 mW, 11 mW/cm^2^, 5 J/cm^2^, 7 min 35 s	Gene expression	Upregulated the expression of mitochondrial genes COX6B2 (complex IV), COX6C (complex IV), PPA1 (complex V), ATP4B (complex V) and ATP5G2 (complex V), ATP5F1 (complex V), NDUFA11 (complex I), and NDUFS7 (complex I).
Goralczyk et al., 2016 [98]	Human umbilical vein endothelial cells	E1: 635 nm, 30 mW, 1066 s, 1.875 mW/cm^2^ E2: 830 nm, 60 mW, 533 s, 3.75 mW/cm^2^ 80 cm^2^ irradiated area, 10 cm distance	TNF-α concentration IL-6 concentration	TNF-α level decreased. LLLT did not cause significant changes in concentration of IL-6 in the endothelial cell culture.
Ayuk et al., 2016 [93]	Human skin fibroblasts	830 nm, 5 J/cm^2^, continuous, 98 mW, 9.1 cm^2^, 10.76 mW/cm^2^, 7 min 43 s	Gene expression profiling	Stimulatory effect on cadherins, integrins, selectins and immunoglobulins.
Ayuk et al., 2018 [94]	Human skin fibroblast cells (WS1)	660 nm, 5 J/cm^2^, continuous, 102 mW, 9.1 cm^2^, 11.23 mW/cm^2^, 7 min 25 s	Cell migration Cell viability Cell proliferation	Wound closure at 24 h as compared to 0 h. Significant increase in cell at 24 h as compared to 0 h. Increase in S-phase and decrease in G2M phase.
**Near-infrared**				
Danno et al., 2001 [36]	Human foreskin keratinocytes; human foreskin microvascular endothelial cells; human newborn foreskin fibroblasts	Halolamps with 0.7–1.3 μm near infrared, 30 mW/cm^2^, 20–60 min at distance of 20 cm	TGF-β1 Matrix metalloproteinase (MMP)-2	TGF-β1 significantly more elevated after irradiation than sham-irradiated controls. Greater increase in MMP-2 was found after irradiation than sham-irradiated controls.
**Polychromatic light emitting diode (LED) energy**				
Vinck et al., 2005 [45]	Chicken embryos fibroblast cultures (*n* = 256)	Green light of 570 nm, continuous mode, 0.1 J/cm^2^, 3 min, 10 mW, once per day for 3 days	Fibroblast survival and proliferation	Significantly higher rate of proliferation in hyperglycemia circumstances after irradiation.
Wu et al., 2015 [91]	Primary human dermal fibroblasts in 180 mM glucose concentration	Organic light-emitting diode, 623 nm peak wavelength; 7 or 10 mW/cm^2^, 0.2, 1 or 5 J/cm^2^	Adenosine triphosphate assay MTS assay CyQuant assay	Increase in total adenosine triphosphate production at both power densities except the power density of 10 mW/cm^2^ and 5 J/cm^2^. Mitochondrial metabolism was significantly higher. Significantly higher cellular proliferation with groups irradiated with 10 mW/cm^2^.

E, Experimental group; C, Control group.

**Table 7:** Characteristics of animal experimental studies.

Reference	(1)	(2)	(3)	(4)	(5)	(6)	(7)	(8)	(9)	(10)
**Pulsed electromagnetic field**										
Callaghan et al., 2008 [19]	Unclear	Yes	Unclear	Unclear	Unclear	Unclear	Unclear	Yes	Yes	Yes
Goudarzi et al., 2010 [20]	Unclear	Yes	Unclear	Unclear	Unclear	Unclear	Unclear	Yes	Yes	Yes
Cheing et al., 2014 [21]	Unclear	Yes	Unclear	Unclear	Unclear	Unclear	Yes	Yes	Yes	Yes
Choi et al., 2016 [22]	Unclear	Yes	Unclear	Unclear	Unclear	Unclear	Unclear	Yes	Yes	Yes
Choi et al., 2018 [23]	Yes	Yes	Unclear	Unclear	Unclear	Yes	Unclear	Yes	Yes	Yes
**Ultrasound**										
Thawer et al., 2004 [24]	Unclear	Yes	Yes	Unclear	Unclear	Unclear	Yes	Yes	Yes	Yes
Mann et al., 2014 [25]	Unclear	Yes	Unclear	Unclear	Unclear	Unclear	Yes	Yes	Yes	Yes
Roper et al., 2015 [26]	Unclear	Yes	Unclear	Unclear	Unclear	Unclear	Unclear	Yes	Yes	Yes
**Shockwave**										
Kuo et al., 2009 [27]	Unclear	Yes	Unclear	Unclear	Unclear	Unclear	Unclear	Yes	Yes	Yes
Zins et al., 2010 [30]	Unclear	Yes	Unclear	Unclear	Unclear	Unclear	Unclear	Yes	Yes	Yes
Yang et al., 2011 [28]	Unclear	Yes	Unclear	Unclear	Unclear	Unclear	Unclear	Yes	Yes	Yes
Hayashi et al., 2012 [29]	Unclear	Yes	Unclear	Unclear	Unclear	Unclear	Unclear	No	Yes	Yes
**Electrical stimulation**										
Smith et al., 1984 [31]	Unclear	Yes	Unclear	Unclear	Unclear	Unclear	Unclear	No	Yes	Yes
Thawer et al., 2001 [32]	Unclear	Yes	Unclear	Unclear	Unclear	Unclear	Unclear	Yes	Yes	Yes
Kim et al., 2014 [33]	Unclear	Yes	Unclear	Unclear	Unclear	Unclear	Unclear	Yes	Yes	Yes
Langoni et al., 2014 [34]	Unclear	Yes	Unclear	Unclear	Unclear	Unclear	Unclear	Yes	Yes	Yes
**Photo energy**										
Yu et al., 1997 [35]	Unclear	Yes	Unclear	Unclear	Unclear	Unclear	Unclear	Yes	Yes	Yes
Danno et al., 2001 [36]	Unclear	Yes	Unclear	Unclear	Unclear	Unclear	Unclear	Yes	Yes	Yes
Reddy et al., 2001 [37]	No	Yes	Unclear	Unclear	Unclear	Unclear	Unclear	Unclear	Yes	No
Stadler et al., 2001 [38]	Unclear	Yes	Unclear	Unclear	Unclear	Unclear	Unclear	Yes	Yes	Yes
AI-Watban and Andres, 2003 [39]	Yes	Yes	Unclear	Unclear	Unclear	Unclear	Unclear	Yes	Yes	Yes
Reddy, 2003 [40]	Unclear	Yes	Unclear	Unclear	Unclear	Unclear	Unclear	Yes	Yes	No
Whelan et al., 2003 [41]	Unclear	Yes	Unclear	Unclear	Unclear	Unclear	Unclear	Yes	Yes	Yes
Byrnes et al., 2004 [42]	No	Yes	Unclear	Unclear	Unclear	Unclear	Yes	Yes	Yes	No
Kawalec et al., 2004 [43]	Unclear	Yes	Unclear	Unclear	Unclear	Unclear	Yes	Yes	Yes	Yes
Maiya et al., 2005 [44]	Unclear	Yes	Unclear	Unclear	Unclear	Unclear	Yes	Yes	Yes	Yes
AI-Watban and Andres, 2006 [46]	Yes	Yes	Unclear	Unclear	Unclear	Unclear	Unclear	Yes	Yes	Yes
Carvalho et al., 2006 [47]	Unclear	Yes	Unclear	Unclear	Unclear	Unclear	Unclear	Yes	Yes	Yes
Rabelo et al., 2006 [48]	Unclear	Yes	Unclear	Unclear	Unclear	Unclear	Unclear	Yes	Yes	Yes
AI-Watban et al., 2007 [49]	Unclear	Yes	Unclear	Unclear	Unclear	Unclear	Unclear	Yes	Yes	Yes
Meireles et al., 2008 [55]	Unclear	Yes	Unclear	Unclear	Unclear	Unclear	Unclear	Yes	Yes	Yes
AI-Watban, 2009 [56]	Unclear	Yes	Unclear	Unclear	Unclear	Unclear	Unclear	Yes	Yes	Yes
AI-Watban et al., 2009 [57]	Unclear	Yes	Unclear	Unclear	Unclear	Unclear	Unclear	Yes	Yes	Yes
Dall Agnol et al., 2009 [58]	Unclear	Yes	Unclear	Unclear	Unclear	Unclear	Unclear	Yes	Yes	Yes
Gungormus and Akyol, 2009 [59]	Unclear	Yes	Unclear	Unclear	Unclear	Unclear	Unclear	Yes	Yes	No
Akyol and Gungӧrmuş, 2010 [60]	Unclear	Yes	Unclear	Unclear	Unclear	Unclear	Yes	Yes	Yes	No
Carvalho pde et al., 2010 [61]	Unclear	Yes	Unclear	Unclear	Unclear	Unclear	Unclear	Yes	Yes	Yes
Chung et al., 2010a [63]	Unclear	Yes	Unclear	No	Unclear	Unclear	Yes	Yes	Yes	Yes
Chung et al., 2010b [62]	Unclear	Yes	Unclear	No	Unclear	Unclear	Unclear	Yes	Yes	Yes
Jahangiri Noudeh et al., 2010 [66]	Unclear	Yes	Unclear	No	Unclear	Unclear	Unclear	Yes	Yes	Yes
Oliveira et al., 2010 [67]	Unclear	Yes	Unclear	Unclear	Unclear	Unclear	Yes	Yes	Yes	Yes
Santos et al., 2010 [68]	Unclear	Yes	Unclear	Unclear	Unclear	Unclear	Yes	Yes	Yes	Yes
Hegde et al., 2011 [69]	Unclear	Yes	Unclear	Unclear	Unclear	Unclear	Unclear	Yes	Yes	Yes
Oliveira et al., 2011 [70]	Unclear	Yes	Unclear	Unclear	Unclear	Unclear	Yes	Yes	Yes	Yes
Peplow et al., 2011 [71]	Unclear	Yes	Unclear	No	Unclear	Unclear	Unclear	Yes	Yes	Yes
Dadpay et al., 2012 [74]	No	Yes	Unclear	Unclear	Unclear	Unclear	Unclear	Yes	Yes	No
Park and Kang, 2012 [89]	No	Yes	Unclear	Unclear	Unclear	Unclear	Unclear	Yes	Yes	No
Peplow et al., 2012 [76]	Unclear	Yes	Unclear	Unclear	Unclear	Unclear	Unclear	Yes	Yes	Yes
Aparecida Da Silva et al., 2013 [77]	Unclear	Yes	Unclear	Unclear	Unclear	Unclear	Unclear	Yes	Yes	Yes
Firat et al., 2013 [86]	Unclear	Yes	Unclear	Unclear	Unclear	Yes	Yes	Yes	Yes	Yes
Franca et al., 2013 [87]	Unclear	Yes	Unclear	Unclear	Unclear	Yes	Yes	Yes	Yes	Yes
He et al., 2013 [79]	Unclear	Yes	Unclear	Unclear	Unclear	Unclear	Yes	Yes	Yes	Yes
Masha et al., 2013 [88]	Unclear	Yes	Unclear	Unclear	Unclear	Unclear	Unclear	Yes	Yes	Yes
Dancáková et al., 2014 [80]	Unclear	Yes	Unclear	Unclear	Unclear	Unclear	Unclear	Yes	Yes	Yes
Kilík et al., 2014 [82]	No	Yes	Unclear	Unclear	Unclear	Unclear	Unclear	Yes	Yes	No
Sharifian et al., 2014 [83]	No	Yes	Unclear	Unclear	Unclear	Unclear	Unclear	Yes	Yes	No
De Loura Santana et al., 2015 [84]	Unclear	Yes	Unclear	Unclear	Unclear	Unclear	Yes	Yes	Yes	Yes
Fekrazad et al., 2015 [92]	Unclear	Yes	Unclear	No	Unclear	Unclear	Yes	Unclear	Yes	Yes
Lau et al., 2015 [85]	Unclear	Yes	Unclear	Unclear	Unclear	Unclear	Unclear	Yes	Yes	Yes
Lau et al., 2015 [90]	Unclear	Yes	Unclear	Unclear	Unclear	Unclear	Unclear	Unclear	Yes	Yes
Wu et al., 2015 [91]	No	Yes	Unclear	Unclear	Unclear	Unclear	Unclear	Yes	Yes	No
de Loura Santana et al., 2016 [95]	Unclear	Yes	Unclear	Unclear	Unclear	Unclear	Yes	Unclear	Yes	Yes
Ranjbar et al., 2016 [99]	Unclear	Yes	Unclear	Unclear	Unclear	Unclear	Yes	Unclear	Yes	Yes
Tatmatsu Rocha et al., 2016 [100]	Yes	Yes	Unclear	Unclear	Unclear	Unclear	Yes	Unclear	Yes	Yes
Denadai et al.,2017 [96]	Yes	Yes	Unclear	Unclear	Unclear	Unclear	Unclear	Unclear	Yes	Yes
Eissa and Salih, 2017 [97]	Yes	Yes	Unclear	Unclear	Unclear	Unclear	Unclear	Unclear	Yes	Yes

Studies fulfilling the criteria of: (1) sequence generation; (2) baseline characteristics; (3) allocation concealment; (4) random housing; (5) investigator blinding; (6) random outcome assessment; (7) assessor blinding; (8) incomplete outcome data addressed; (9) free of selective outcome reporting; and (10) free of other source of bias.

## Abbreviations

BPEs	biophysical energies
ES	electrical stimulation
PEMF	pulsed electromagnetic field
ECSW	extracorporeal shockwave
LLL	low-level laser therapy
US	ultrasound
LED	light emitting diode
NIR	infrared
E	experimental group
C	control group

## Appendix A

### Detailed search strings

Basic search was combined with searches for interventions by adding the search term AND.

### Basic search

(Diabetes Mellitus [MeSH]) OR (Diabetes Mellitus) OR (Diabetes) OR (Diabetic) OR (Diabetes Mellitus, Type 2)) OR (Diabetes Complications [MeSH]) AND (ulcer [MeSH]) OR ((Foot ulcer) OR (diabetic foot) OR (wound) OR (wound healing [MeSH]) OR (wounds and injuries [MeSH]).

### Model

(Animal) OR (Animals [MeSH]) OR (mouse) OR (Mice [MeSH]) OR (murine) OR (Rats [MeSH]) OR (rodent) OR (Hamster) OR (Cricetulus [MeSH]) OR (Rabbits [MeSH]) OR (Guinea pigs [MeSH]) OR (Swine [MeSH]) OR (dog) OR (porcine) OR (Sprague-Dawley) OR (Transgenic) OR (Sheep [MeSH]) OR (pig) OR (*In Vitro* [MeSH]) OR (*In vivo*) OR (Cells [MeSH]) OR (macrophages) OR (fibroblasts) OR (Adenosine triphosphate) OR (Collagen).

### Electrical stimulation

(Physical therapy modalities [MeSH]) OR (Electric stimulation therapy [MeSH]) OR (Electric* therapy) OR (Microamperage stimulation) OR (Low intensity direct current) OR (High voltage) OR (electrotherapy) OR (direct current) OR (microcurrent).

### Electromagnetics

(Electromagnetic*) OR (Electromagnetic Fields [MeSH]) OR (Magnetic Field Therapy [MeSH]) OR (Pulsed electromagnetic therapy) OR (diathermy) OR (shortwave).

### Phototherapy

(Ultraviolet rays [MeSH]) OR (Lasers [MeSH]) OR (Laser Therapy [MeSH]) OR (Laser Therapy, Low-Level [MeSH]) OR (MIRE) OR (monochromatic infrared energy) OR (Phototherapy [MeSH]) OR (Infrared Rays [MeSH]) OR (Anodyne) OR (near infrared) OR (near-infrared).

### Ultrasound

(Ultrasound [MeSH]) OR (Ultrasonic Therapy [MeSH]) OR (Ultrasonic Therap*) OR (ultrasonic).

### Extracorporeal shockwave therapy

(extracorporeal shockwave) OR (shockwave).

### Filter

NOT (“review” [Publication Type]) OR (review literature as topic [MeSH]) OR (reviews).
